# The complete chloroplast genome of *Alniaria alnifolia* (Siebold and Zucc.) Rushforth, 2018

**DOI:** 10.1080/23802359.2022.2116957

**Published:** 2022-09-07

**Authors:** Mei Yu, Jian-wen Bu, Biao Han, Dan Liu, Chang Lu, Lei Wang

**Affiliations:** a College of Food Science and Engineering, Shandong Agriculture and Engineering University, Ji’nan, P. R. China; b Key Laboratory of National Forestry and Grassland Administration Conservation and Utilization of Warm Temperate Zone Forest and Grass Germplasm Resources, Shandong Provincial Center of Forest and Grass Germplasm Resources, Ji’nan, P. R. China

**Keywords:** *Alniaria alnifolia*, Illumina, Rosaceae, phylogenetic

## Abstract

*Alniaria alnifolia* (Siebold and Zucc.) Rushforth, 2018 (alternative name: *Sorbus alnifolia*) belongs to the genus *Alniaria* of the family *Rosaceae* and is widely distributed in northern China, Korea, and Japan. It is an essential resource used in the construction, pharmaceuticals, and food industries. It is also used to treat various diseases, such as fever, hyperglycemia, rash, asthma, bronchitis, constipation, leprosy, anemia, and other skin ailments. In this study, we sequenced a sample of *A. alnifolia* and determined its complete chloroplast genome. The chloroplast genome of *A. alnifolia* has a circular structure with a length of 159,855 bp, which includes a small single-copy region (19,409 bp), a large single-copy region (87,628 bp), and two inverted repeats (26,409 bp). The sequence had 130 genes, including 85 protein-coding genes, eight rRNA genes, and 37 tRNA genes, and the overall GC content was 36.6%. The genes *trn*K-UUU, *rps*16, *trn*G-UCC, *atp*F, *rpo*C1, *trn*L-UAA, *trn*V-UAC, *pet*B, *pet*D, *rpl*16, *rpl*2, *ndh*B, *trn*I-GAU, *trn*A-UGC, and *ndh*A contained one intron; genes *clp*P and *ycf*3 contained two introns. Phylogenetic results showed that *A. alnifolia* had the closest relationship with *Sorbus folgneri* (MK161058).

*Alniaria alnifolia* (Siebold and Zucc.) Rushforth, 2018 is the latest scientific name for *Sorbus alnifolia* (Siebold and Zucc.) K. Koch (Schoch et al. 2020), popularly known as the alder-leafed white beam or Korean white beam. This plant belongs to the genus *Alniaria* in the family *Rosaceae*, is widely distributed in northern China, the Korean Peninsula, and Japan, and is an important resource in the construction, pharmaceutical, and food industries. The bark of plant species belonging to the *Alniaria* genus is believed to possess strong therapeutic potential for neurological disorders, such as stroke and neurological pain (Cheon et al. 2017). Therefore, it is necessary to determine the structure and gene content of the complete chloroplast genome of *A. alnifolia* and to confirm the phylogenetic relationships within Rosaceae. This could be conducive to further research of *A. alnifolia* in other fields.

The sample of *A. alnifolia* was collected from Mengshan Mountain, Linyi, Shandong Province, China (N35°33′32″, E117°50′41″) and stored at the Shandong Provincial Center of Forest and Grass Germplasm Resources (barcode SDF1005909, Lei Wang, Email: stoawang@126.com). This study was approved by the Shandong Agriculture and Engineering University and Shandong Provincial Center of Forest and Grass Germplasm Resources and complies with the National Wild Plant Protective Regulations. Permission for sample collection was granted by the administrative committee of the Mengshan Scenic Area in Pingyi County, Shandong Province.

Fresh leaves (0.5 g) were extracted using the improved cetyltrimethylammonium bromide (CTAB) method (Doyle and Doyle 1987), and the DNA quality was detected by agarose gel electrophoresis using a microspectrophotometer (Nanodrop-2000). The detection results showed that the DNA was not degraded, its concentration and total amount were 2.6 ng/µl and 0.2 µg, respectively. After quality inspection, the total genomic DNA was constructed in a sequencing library with a 350 bp insert using the NexteraXT DNA library preparation kit, and double-terminal sequencing was performed on the library using the Illumina Novaseq 6000 sequencing platform. After obtaining the raw sequence data, NGS QC Tool-Kit software was used to filter out low-quality sequences to obtain 1.8 Gb (clean reads) high-quality data (Q20 = 97.22%, Q30 = 92.08%), and the average sequencing depth of the chloroplast genome reached 1781 X. SPAdes V3.11.0 software (Bankevich et al. 2012) was used for *de novo* assembly of the filtered reads to obtain the complete chloroplast genome. It was annotated using PGA software (Qu et al. 2019) with *Sorbus vilmorinii* (MK920285) as a reference genome. Finally, we submitted the assembled complete chloroplast genome sequence data to GenBank under accession numbers MZ145061 and SRA (SRR14663461) submitted to NCBI under BioProject No. PRJNA732298.

The chloroplast genome of *A. alnifolia* had a typical quadripartite structure with a size of 159,855 bp. It contained a large single-copy region (LSC:87,628 bp), a small single-copy region (SSC:19,409 bp), and two inverted repeats (IRs:26,409 bp), with an overall GC content of 36.6%. There were 130 genes, including 85 protein-coding genes, 37 tRNA genes, and eight rRNA genes. The total length of the protein-coding genes was 80,415 bp (50.14%) with a GC content of 37.59%. Furthermore, the total RNAs (including tRNA and rRNAs) were 11,839 bp in length (7.41%), with a GC content of 55.06%. Among the 130 genes, 15 (*trn*K-UUU, *rps*16, *trn*G-UCC, *atp*F, *rpo*C1, *trn*L-UAA, *trn*V-UAC, *pet*B, *pet*D, *rpl*16, *rpl*2, *ndh*B, *trn*I-GAU, *trn*A-UGC, and *ndh*A) contained one intron, and two genes *(clp*P and *ycf*3) contained two introns, whereas *rps*12 showed trans-splicing.

To determine the phylogenetic relationship between *A. alnifolia* and other members of the *Rosaceae* family, we selected and downloaded 12 complete chloroplast genome sequences belonging to the family Rosaceae from NCBI and aligned them with *A. alnifolia* using Mafft 7.473 (Katoh and Standley 2013) with the FFT-NS-2 strategy. After obtaining the aligned file, we used a model finder to select the TVM + F+I + G4 model (Kalyaanamoorthy et al. 2017) and used IQtree 2.0 (Minh et al. 2020) to construct a phylogenetic tree (Figure 1) with a bootstrap value of 1000 using the maximum-likelihood method. During the ML tree construction, the complete chloroplast genome of *Crataegus kansuensis* (NC_039374) was used as an outgroup. In our results, *A. alnifolia* showed the closest relationship with *Sorbus folgneri* (MK161058), which was consistent with the findings of Qiu et al. (2019), where they used 17 complete chloroplast genomes to construct the ML phylogenetic tree. However, the chloroplast genome of *A. alnifolia* used in their study was incomplete. The results of the phylogenetic analysis strongly support the findings of Qiu et al. (2019). Moreover, there are many evolutionary divergences within the genus *Sorbus*. The two groups in this study consisted of group 1 and group 2. In group 1, *Sorbus aria, Sorbus chamaemespilus,* and *Sorbus torminalis* had a close relationship; while in group 2, which had nine species, including *Sorbus vilmorini,* did not have a very close relationship with other species. The relationships of *Sorbus commixta, Sorbus amabilis, Sorbus tianschanica, Sorbus setschwanensis,* and *Sorbus insignis* were closer as compared to *S. vilmorinii.*

**Figure 1.**
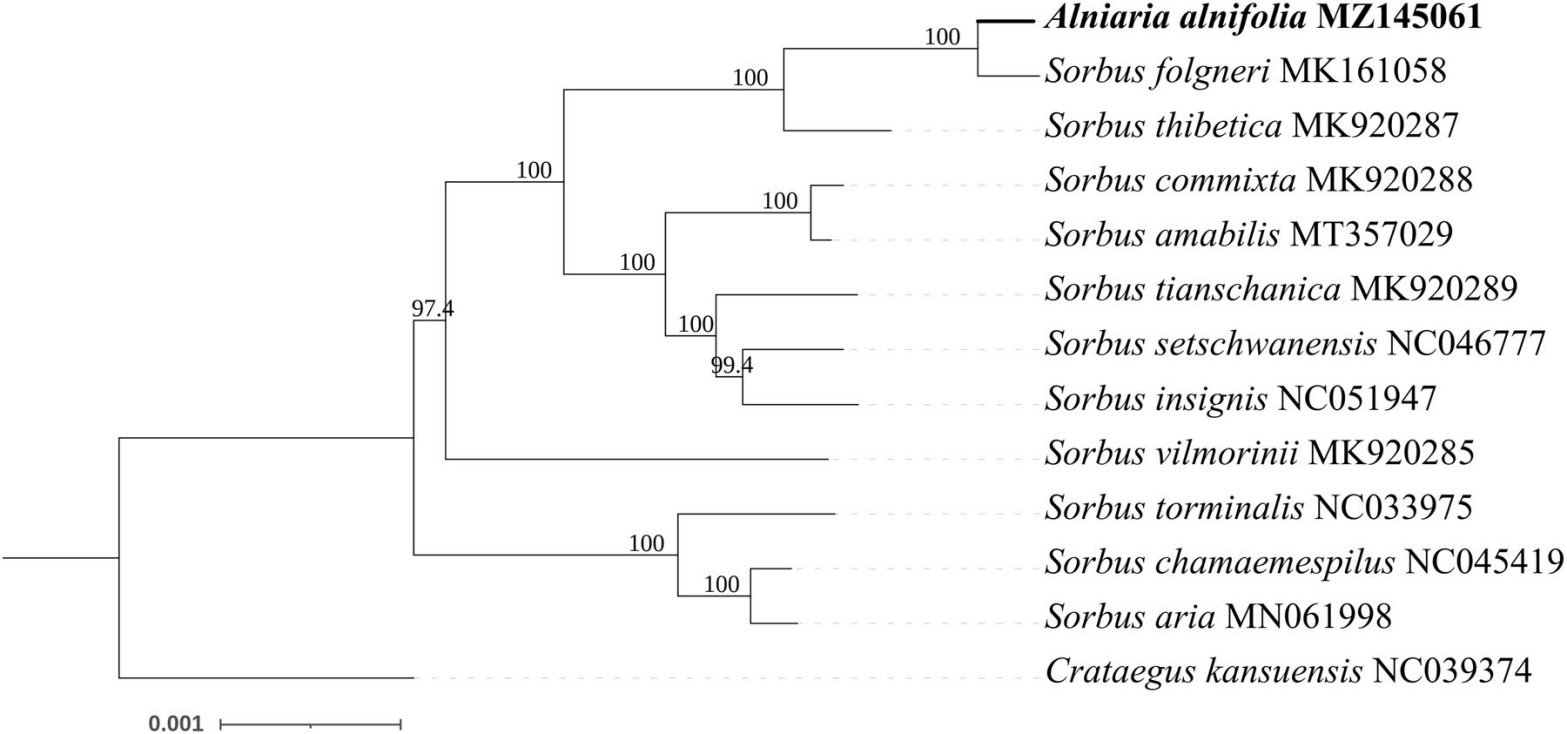
Maximum-likelihood phylogenetic tree for *A. alnifolia* based on 13 complete chloroplast genomes.

## Data Availability

The genome sequence data that support the findings of this study are openly available in GenBank (https://www.ncbi.nlm.nih.gov/) under accession no. MZ145061. The associated BioProject, SRA, and Bio-Sample numbers are PRJNA732298, SRR14663461, and SAMN19316646, respectively.
